# Regulation of Brown Adipose Tissue Activity by Interoceptive CNS Pathways: The interaction between Brain and Periphery

**DOI:** 10.3389/fnins.2017.00640

**Published:** 2017-11-16

**Authors:** Otto Muzik, Vaibhav A. Diwadkar

**Affiliations:** ^1^Departments of Pediatrics, Wayne State University School of Medicine, Detroit, MI, United States; ^2^Radiology, Wayne State University School of Medicine, Detroit, MI, United States; ^3^Psychiatry and Behavioral Neurosciences, Wayne State University School of Medicine, Detroit, MI, United States

**Keywords:** CNS thermoregulation, human brown adipose tissue, functional MRI, FDG PET, variability of BAT mass

## Abstract

To maintain thermal homeostasis, specific thermogenic tissues are under the control of central thermoregulatory networks that regulate the body's response to thermal challenges. One of these mechanisms involves non-shivering thermogenesis in brown adipose tissue (BAT), which is activated in cold environments in order to defend the body against physical damage as a result of hypothermia. The objective of our study was to assess the interaction between CNS thermoregulatory pathways and sympathetic innervation in BAT during a cold exposure paradigm. Our results show that an innocuous whole-body cooling paradigm induces significant differences in fMRI BOLD signal at the location of the right anterior insula and the red nucleus/substantia nigra region, between lean subjects with high levels of sympathetic innervation in supraclavicular BAT (BAT+ group), and subjects with low levels of sympathetic innervation (BAT− group). Specifically, results indicate significantly larger fMRI BOLD signal changes between periods of cooling and warming of the skin in the BAT+ (as compared to BAT−) group at the location of the right anterior insula. In contrast, the BAT+ group showed significantly smaller fMRI BOLD signal changes in the midbrain between periods of skin cooling and warming. Our findings are consistent with a hierarchical thermoregulatory control system that involves the initiation of inhibitory signals from the right anterior insula toward midbrain areas that normally exert tonic inhibition on the medullary raphe, from where BAT is directly innervated. Our data suggests that exposure to cold elicits differential neuronal activity in interoceptive regulatory centers of subjects with high and low level of sympathetic innervation. As a result, the variability of cold-activated BAT mass observed in humans might be, in part, yoked to different sensitivities of interoceptive cortical brain areas to skin temperature changes.

## Introduction

A fundamental homeostatic function of the central nervous system (CNS) is to protect the body against damage caused by hypothermia. One of several thermoregulatory mechanisms contributing to the generation of heat in order to maintain thermal homeostasis is the activation of peripheral brown adipose tissue (BAT) by the sympathetic nervous system (SNS). The presence of BAT in supraclavicular regions of humans that can be activated by cold exposure has been well documented (Cypess et al., 2009; Virtanen et al., 2009). BAT thermogenesis is governed by brain regions involved in both thermal interoception (Craig, 2003; Fealey, 2013) as well as in the regulation of body temperature (Nakamura and Morrison, 2008a) and energy balance (Richard, 2015). These mechanisms have been well studied in animal models: work in rodents has identified neural circuits in the brainstem that include the parabrachial complex, preoptic area of the hypothalamus and the raphe pallidus as critical regulators of the sympathetic innervation of BAT (Morrison and Nakamura, 2011; Bartness and Ryu, 2015), but human *in vivo* studies have been hampered by technical challenges of co-acquiring neuroimaging and physiological signals during the application of whole body stress. We have previously studied sympathetic innervation in human supraclavicular BAT (both at rest and during cold exposure) using PET/CT imaging with 11C-meta-hydroxyephedrin, a structural analog of the neurotransmitter norepinephrine (Muzik et al., 2017). The results of this study demonstrated that differences in cold-activated BAT mass between subjects are related to the level of their peripheral sympathetic innervation. Moreover, our data showed that the amount of cold-activated BAT mass (determined based on increased glucose consumption) is proportional to the degree of sympathetic control exerted by the CNS on peripheral BAT. In addition to these molecular imaging studies of BAT, we have recently begun to detail fMRI estimated activations (and time series of activations) of CNS pathways in response to decreases in skin temperature (Muzik and Diwadkar, 2016). These studies have shown that, in addition to the activation of homeostatic nuclei within the brainstem that initially sense (Craig, 2002) and subsequently mediate autonomic CNS responses to peripheral inputs (Satinoff, 1978; Egan et al., 2005), these signals also projected to multiple cortical areas, such as the insular cortex, the orbitofrontal cortex, the anterior cingulate and the posterior parietal somatosensory cortex, where subjective feelings are generated. Multiple lines of evidence across mammalian studies imply that this value-generating network generate hedonic valence that subsequently guides behavior (Craig, 2002; Diwadkar et al., 2014). However, the relationship between activation within this network and the level of peripheral sympathetic innervation (inferred based on the amount of cold-activated BAT) in humans has never been performed.

Understanding this relationship is of foundational interest. For unknown reasons the amount of cold-activated BAT in humans is highly variable, as is the subjective experience of cold. However, if these variations are linked to differences in the level of sympathetic innervation governed by the CNS, it is plausible that: (a) induced experimenter-controlled changes in thermoregulatory defense will induce differential changes in BAT and (b) sub-groups with differential effects in cold-activated BAT will also be characterized by systematic differences in the range of their fMRI responses to cooling and rewarming. These motivations drove the current multi-modal imaging study in which we acquired within-participant data using two modalities (PET and fMRI).

We first employed 18F-fluorodeoxyglucose (FDG) PET imaging to stratify subjects into groups according to their cold-activated BAT mass, an indicator of peripheral sympathetic innervation levels (Muzik et al., 2017). Subsequently all the participants were subjected to an identical oscillating thermal challenge using fMRI, wherein we assessed the time series and the difference in amplitude of the responses in previously characterized cortical and sub-cortical regions (Diwadkar et al., 2014; Muzik and Diwadkar, 2016) during whole-body cooling and rewarming. This focus allowed us to estimate the range of neuronal activations (Rees et al., 2000) based on the modeled time series oscillations in a previously identified brain network (Muzik and Diwadkar, 2016) associated with thermal processing. As a result, CNS interoceptive as well as thermoregulatory brain pathways could be assessed relative to measured sympathetic innervation levels observed in BAT. The strength of this approach is that it relates the interpretation of the fMRI data (acquired in the CNS) to a specific physiological context (acquired in the periphery).

## Materials and methods

### Subjects

A total of 20 lean healthy subjects (10M/10F, 23.3 ± 3.8 years, BMI = 18.5–24.9 kg/m^2^) subjects were studied (Table 1). Stature was measured to the nearest cm and weight was measured to the nearest 0.5 kg, following standard procedures. The body mass index (BMI) was calculated as weight/height^2^ (kg/m^2^). Percent body fat (%) was calculated from the sum of skinfold measurements at the biceps, triceps, subscapular and suprailiac sites using Lange calipers. The lean body mass (LBM; kg) was subsequently calculated as body weight less fat mass. This study was carried out in accordance with the recommendations and approval of the Wayne State University Human Investigation Committee. All subjects gave written informed consent in accordance with the Declarations of Helsinki. The protocol was approved by the Wayne State University Institutional Review Board.

**Table 1:** Descriptive statistics of subjects in the high-BAT (BAT+) and low-BAT (BAT−) group.

**Parameter**	**All subjects**	**BAT±**	**BAT–**	***P***
Subjects	20 (10F/10M)	12 (6F/6M)	8 (4F/4M)	
Age (years)	23.3 ± 3.8	24.1 ± 4.0	22.5 ± 3.5	NS
Height (cm)	172 ± 12	170 ± 13	175 ± 12	NS
Weight (kg)	69 ± 16	65 ± 16	76 ± 17	NS
BMI (kg/m^2^)	23.0 ± 2.6	22.0 ± 2.1	24.8 ± 2.4	0.03
Body Fat (%)	25.4 ± 5.8	25.3 ± 6.3	25.5 ± 5.2	NS
BAT mass (g)	68 ± 49	96 ± 37	16 ± 4	<0.01
BAT SUVmax	16.2 ± 8.8	19.5 ± 8.0	7.2 ± 1.7	<0.01
BAT Metab. Act. (g)	468 ± 450	609 ± 451	81 ± 22	<0.01
DEE rest (kcal/day)	1,502 ± 366	1,401 ± 375	1,654 ± 313	NS
DEE cold (kcal/day)	1,697 ± 427	1,745 ± 473^*^	1,625 ± 411	NS
Δ WB DEE (%)	10.5 ± 21.3	17.1 ± 16.5	−2.8 ± 23.4	NS

* *p < 0.01 rest vs. cold*.

### Study design

All subjects underwent 18F-fluorodeoxyglucose (FDG) PET imaging during mild cold condition in order to assess the presence of cold-activated supraclavicular BAT. Whole body changes in daily energy expenditure (DEE) between the cold and thermoneutral condition were assessed using indirect calorimetry and related to changes in BAT DEE. In addition, to assess functional brain responses to cold stress, all subjects underwent a functional MRI (fMRI) scan paradigm. During fMRI, subjects underwent a carefully manipulated oscillating whole body temperature paradigm. The paradigm induced periods of mild hypothermia which was interspersed by periods designed to return the body to basal core temperature. The fMRI study was performed on a separate day and within a week of the PET scan. Finally, all studies were performed during the spring/summer months, thus differential activation of BAT due to outside day temperature is unlikely.

A histogram of cold-activated BAT mass showed two distinct peaks with maxima at 10–20 g and at 90–100 g. To account for the bimodal distribution, subjects were divided into two groups (High-BAT or BAT+ and Low-BAT or BAT−), with the threshold separating the two groups set to 20 g of activated BAT (see Table 1). All participants were screened for medical history and metabolic status, as assessed on the basis of routine laboratory tests and measured blood pressure.

**Figure 1:**
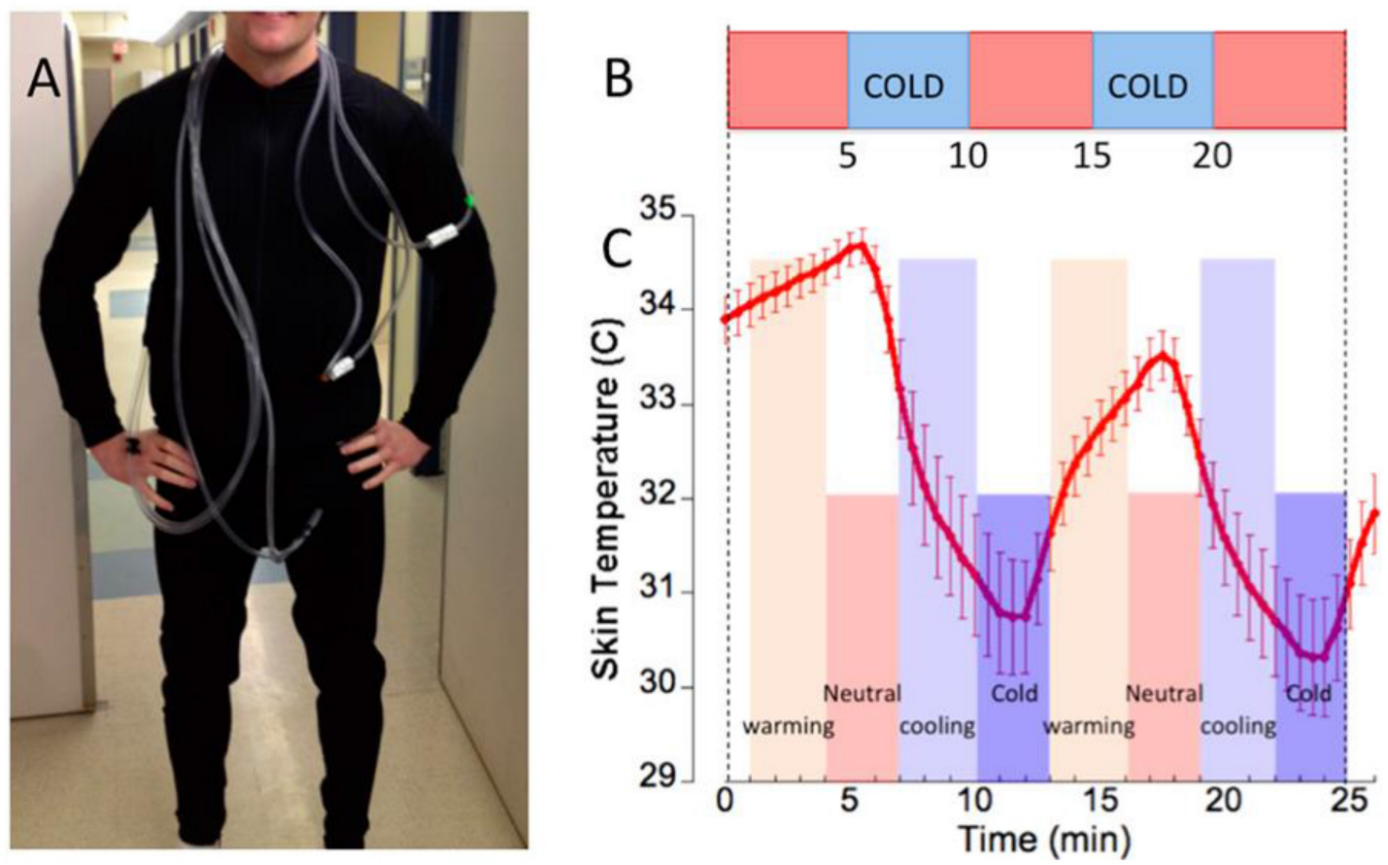
**(A)** Subject dressed in the tube suit covering the arms to the wrists, the legs to the ankles and the torso. **(B)** The bar depicts the stimulus (study paradigm) consisting of two 5-min cooling periods (blue) interspersed between neutral temperature background (orange), resulting in average skin temperature oscillations (error bars ± s.d.) shown below. **(C)** From the skin temperature curve we derived two paired classes of epoch windows representing warming vs. cooling (light orange/light blue) and neutral vs. cold (dark orange/dark blue). Epoch classes used in the comparative analyses are denoted.

Mild cold exposure during each of the PET and fMRI acquisitions was applied using a specialized whole-body garment described in our previous studies (Muzik and Diwadkar, 2016). In short, the garment incorporates a network of small-diameter plastic tubing (Allen Vangard, Inc., Ottawa, CA) (see Figure 1A) through which temperature-controlled neutral (31–34°C) or cold water (15–17°C) is circulated from two separate water reservoirs. Skin temperature was monitored using a GaAs crystal sensor located at the tip of an optical fiber cable (OpSense, Inc., Quebec City, CA), which allows accurate measurement of temperature (to within 0.1°C) even in the MR scanner. The sensor was taped to the skin at the location of the left rib cage. This location was selected on the basis of proximity to important anatomical features (close to the pulmonary blood vessels which are possibly the most representative sites for core body temperature) and the ability to consistently place the sensors based on the anatomical landmark. Subjects were closely monitored during the cold exposure period for signs of shivering. In addition to physiological monitoring of skin temperature, subjects reported every 5 min about his/her subjective feeling of cold. If shivering was likely to occur based on the subject's self-report, the water temperature in the garment was raised. Post-experimental debriefing verified that all subjects experienced the cold condition as a pain-free cold sensation without substantial discomfort.

### PET data acquisition

All subjects were scanned using a GE Discovery STE PET/CT scanner in 3D mode following a ≥6 h fasting period. One hour after the start of cooling, the FDG tracer (150 MBq) was injected intravenously, and following a 50 min uptake period (during which cooling was maintained), a four bed-position PET/CT scan was acquired, covering the torso from the shoulder to the lower abdomen. All PET images were corrected for attenuation as well as tracer decay and reconstructed using the OSEM iterative reconstruction, yielding a final spatial resolution of ~7 mm full-width-at-half-maximum (FWHM).

Activated BAT was defined as tissue areas that were more than 5 mm in diameter and had a minimal standard uptake value [SUV, defined as tracer concentration in MBq/cc normalized to injected activity (MBq) per weight (g)] of FDG of at least 2.0. This cutoff represented more than 2 SD above the maximal SUV seen in typical depots of white adipose tissue (Williams and Kolodny, 2008). BAT volume was determined by thresholding both the CT image volume (−250 < HU < −50) and the FDG volume (SUV > 2.0) and then applying the logical AND operation to the 2 masks, followed by removal of all areas that were smaller than 0.125 cm^3^. The final BAT ROI was chosen at the location of the largest contiguous group of voxels that survived the masking operation. In case that no voxels survived the masking operation (no BAT activation), a volume of ~10 cm^3^ (1.5 × 1.5 × 4.0 cm^3^ ROI) was selected at a typical location of supraclavicular BAT. The volume of BAT ROIs (cm^3^) was converted into weight (g) by assuming a density of 0.90 g/cm^3^.

### MRI procedure

Structural and functional MRI data acquisition was performed on a 3T Siemens Verio system using a 12-channel volume head coil. Initially, a 3D T1-weighted anatomical MRI sequence was acquired (TR: 2,200 ms, TI: 778 ms, TE: 3 ms, flip-angle = 13°, FOV: 256 × 256 mm^2^, 256 axial slices of thickness = 1.0 mm, matrix = 256 × 256, scan-time = 5 min 22 s). Subsequently, a gradient echo EPI fMRI data acquisition was conducted (TR: 2.6 s, TE: 29 ms, FOV: 256 × 256 mm^2^, acquisition matrix: 128 × 128, 36 axial slices, voxel dimensions: 2 × 2 × 3 mm^3^). These parameters allow acquisition of fMRI data with high *in vivo* spatial resolution. The total study time was ~1 h.

The fMRI paradigm consisted of 600 images (*TR* = 2.6 s) resulting in a sequence of 26 min duration blocked into five 5-min epochs (Muzik and Diwadkar, 2016). The epochs alternated between the neutral and cold stimulus (Figure 1B). As shown in the figure, the alternating stimulus induced skin temperature oscillations in an ~4°C (~7°F) range, and this decrease from baseline (which was determined as 34 ± 1.3°C during the time when the structural T1-weighted image was acquired and prior to water being circulated through the tube suit) is notable, given the relatively short duration of cold exposure (5 min). The relatively short time duration of cold exposure (5 min) allows the perception of a “cold” stimulus in the absence of pain.

The resulting skin temperature curve (Figure 1C) is characterized by two distinct skin-temperature epochs: (a) A dynamic gradient associated with **cooling** and **warming** (or return to neutral) that reflects high rates of skin temperature change in response to the stimulus and (b) periods when skin temperature remains relatively stable (**cold** or **neutral**), most likely reflecting physiological adaptation. These distinct epochs constituted separable physiological predictors of the BOLD response, and were employed to construct epochs of interest for fMRI analyses. Each epoch was modeled with a temporal radius of 1.5 min centered at either (a) points of the highest rates of skin temperature change (in the negative or positive direction) or (b) at points of relatively constant skin temperature (at both neutral and cold condition). Thus, in each participant these first level models estimated neuronal responses during (relatively) rapid skin temperature transitions when thermoregulatory demands were maximal, separately from periods of relatively stable skin temperature that reflected adaptation following temporary relaxation of the stimulus.

### Measurement of whole body indirect calorimetry

Measurement of energy expenditure (kcal/day) under resting (prior to cold exposure) and cold (90 min into cold exposure) conditions was performed using the MedGraphics VO2000 Portable Metabolic Testing System (St. Paul, MN). For both conditions, subjects were measured while lying in a relaxed position, in a fasting state (≥6 h). A neoprene face masks was fitted for each subject and heart rate (HR; beats/min), oxygen consumption (VO_2_; l/min), carbon dioxide production (VCO_2_; l/min) and respiratory quotient (RQ; VCO_2_/VO_2_) were all measured for 10 min. Whole body energy expenditure was calculated from VO_2_ and RQ values using the BreezeSuite software (Version 6.0; MedGraphics). Energy expenditure was averaged over 8 min (initial 2 min to reach steady state were discarded) and then multiplied with a factor 1,440 min/day, yielding a daily energy expenditure (DEE, kcal/d).

#### Statistical analysis

##### PET data

PET data are reported as mean ± SD and all analyses were performed in SPSS (v. 21). Normally distributed continuous variables were compared between the BAT+ and BAT− groups using an independent sample *t*-test and correlation between variables were assessed using Pearson's r. All reported *p*-values are two-tailed and values less than 0.05 were considered to indicate statistical significance.

##### fMRI data

The fMRI images were analyzed using SPM8 (Wellcome Department of Cognitive Neurology, Institute of Neurology, London, UK) (Friston et al., 2007). In all analyses, the first four images were discarded to account for EPI equilibration effects. The remaining images in the sequence were realigned to correct for head movements, corrected for slice timing, and subsequently spatially normalized according to the transformation matrix derived between the coregistered (to the mean EPI sequence image) T1-weighted image volume and the MNI template brain. The images were then smoothed spatially with a 3D Gaussian kernel of 6 mm FWHM and re-sampled (2 × 2 × 2 mm^3^). To remove low-frequency scanner drift, a high-pass filter (cutoff 1/128 s) was applied. Subsequently, an established SPM analysis pipeline was applied, as reported previously (Muzik and Diwadkar, 2016). In short, data were modeled voxel-wise, applying a general linear model (GLM) based on a boxcar waveform (according to the previously described epochs modeled from skin temperature data) and convolved with the canonical hemodynamic response function. The confounding effect of global signal intensity was removed using proportional scaling. The first-level analysis included correction for within-scanner motion by means of 6 realignment parameters as regressors, which were derived from the initial realignment step.

Following data preprocessing, variations in fMRI responses during the different epochs were modeled at the first level using pair-wise directional contrasts. Separate contrasts were used for fMRI responses associated with cooling relative to warming, and periods of cold relative to periods of neutral skin temperature (see Figures 1B,C). These individual contrast images were then submitted to a second-level random-effects analysis (Turner et al., 1998), to assess group differences during the various epochs. In order to determine whether gender exerts a significant influence on sympathetic activation of supraclavicular BAT during cold exposure, we initially performed a comparison between males and females within each particular group (BAT+ or BAT−). As no significant regional differences between males and females were determined within each group, the data was collapsed over gender, resulting in the two groups (BAT+ and BAT−). Activations were assessed in the well-established thermoregulatory-interoceptive network that consists of the brainstem, insula, anterior cingulate cortex, orbitofrontal cortex, posterior parietal cortex and the hypothalamus (Craig, 2002; Diwadkar et al., 2014; Muzik and Diwadkar, 2016). All analyses were constrained respecting the relative homogeneity of function within the regions of interest. Significant clusters *within each region* were identified using AlphaSim (Ward, 2000), by estimating the minimum cluster extent sufficient for activated clusters to be rejected as false positive (noise-only) clusters. This approach performs a Monte Carlo alpha probability simulation, by means of computing the probability of a random field of noise (after taking into account the spatial correlations of voxels based on the image smoothness within each region of interest estimated directly from the data set) to produce a cluster of a given size, after thresholding the noise at a specific level. The advantage of this method is that it uses a combination of both cluster size and probability thresholding, believed to be superior to the application of one single voxel probability threshold alone in achieving the desired overall significance level (Ward, 2000). The underlying concept asserts that true areas of activation will tend to occur over contiguous voxels within a region of relative functional homogeneity, whereas noise has a much lower tendency to form activation clusters (Muzik and Diwadkar, 2016). Because recent reports have questioned the significance of activation foci based on cluster-defining thresholds (Eklund et al., 2016), all significant activation clusters were subsequently used as masks in order to extract the fMRI responses at each sampled time point across the whole study in each subject. Specifically, for each activation mask and subject, the first eigenvariate from the modeled fMRI responses time sequence was extracted and subsequently averaged over subjects, yielding a time-series for each group. These time series' represent the % deviation from the mean fMRI signal value and were visually assessed for significance during the appropriate epoch. Activation peaks were reported in Talairach space, following conversion of voxel coordinates in MNI space to Talairach space (Lancaster et al., 2007). Finally, Brodmann areas were reported where appropriate.

## Results

### High- and low-BAT group based on FDG PET

**Figure 2:**
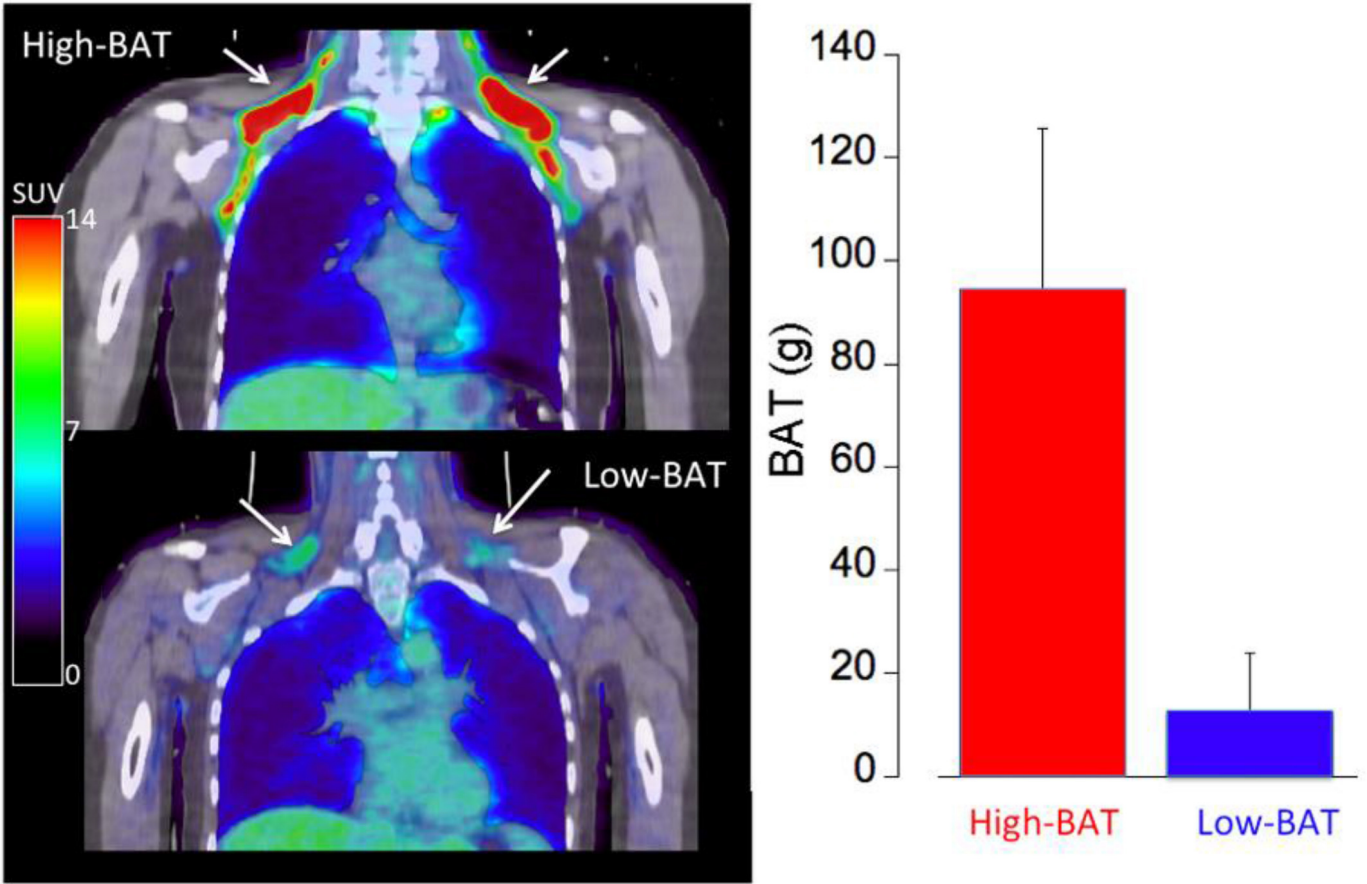
Representative FDG PET/CT images of a subject with large amount of cold- activated supraclavicular BAT mass (BAT+ group, **top left**) and a subject with low amount of cold-activated BAT mass (BAT−, **bottom left**). A standard uptake value (SUV) of > 2.0 was assumed to represent cold-activated BAT at the location of adipose tissue (HU < −50). The graph on the right shows the amount of cold-activated BAT mass in the BAT+ (red) and BAT− (blue) groups.

The study population consisted of lean (BMI = 23.0 ± 2.6 kg/m^2^) young (23.3 ± 3.8 years of age) healthy controls (10M/10F) (Table 1). We determined a wide variability in cold-activated BAT ranging from 0 to 182 g, characterized by a bimodal distribution. Following cold exposure, 60% of the subjects showed relatively high BAT activation (~100 g, High-BAT group or BAT+), whereas 40% of the subjects displayed low BAT activation (<20 g, Low-BAT group or BAT−) (see Figure 2). According to group selection, the SUVmax determined in supraclavicular BAT of the BAT+ group was significantly higher than that measured in the BAT− group (19.5 ± 8.0 vs. 7.2 ± 1.7; *p* < 0.01). Overall, we determined a significant decrease in heart rate (HR: −5.8 ± 4 bpm) and significant increase in mean arterial pressure (MAP: 11 ± 8 mmHg) during cold exposure. However, there was no significant difference with respect to HR and MAP between the BAT+ and BAT− groups.

**Figure 3:**
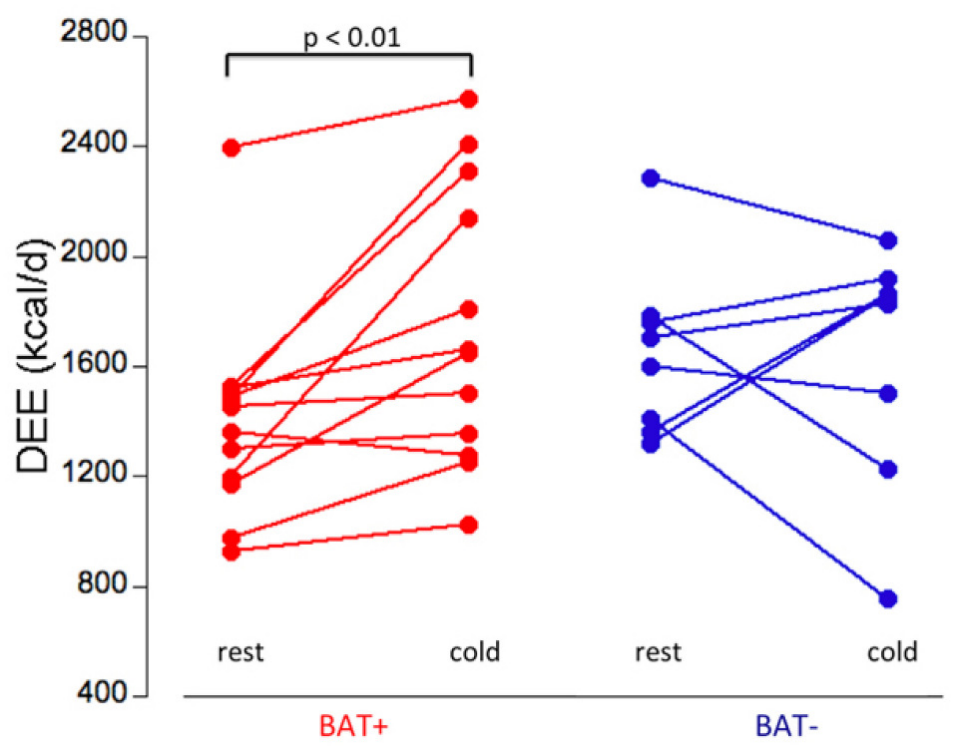
Daily energy expenditure (DEE, kcal/day) at rest and during cold exposure condition in the BAT+ (red) and BAT− (blue) groups. In the BAT+ group, DEE at cold was significantly higher than at rest (paired *t*-test, *p* < 0.01). No significant difference between rest and cold condition was determined in the BAT− group. Moreover, no significant difference in DEE was determined between the groups at either rest or cold condition.

The two groups showed different responses in their daily energy expenditure (DEE, kcal/day) to mild cold exposure. In the BAT+ group the baseline DEE was lower than in the BAT− group, however during mild cold exposure the DEE increased in the BAT+ group by about 17% (from 1,401 ± 375 to 1,745 ± 473 kcal/day, *p* < 0.01), whereas it remained on average virtually constant (−2.8%) in the BAT− group (change 1,654 ± 313 to 1,625 ± 411 kcal/day) (Table 1, Figure 3). Moreover, while absolute changes in DEE were always positive in the BAT+ group (range 48–783 kcal/day, mean = 340 ± 380 kcal/day), DEE changes in the BAT− group varied considerably (range −496 to 538 kcal/day, mean = −29 ± 361 kcal/day).

Finally, the respiratory quotient (RQ = VCO_2_/VO_2_) was marginally lower in the BAT+ as compared to the BAT− group (0.85 ± 0.06 vs. 0.91 + 0.11; *p* = 0.16), suggesting greater levels of resting fat oxidation in this group (McArdle et al., 2001). RQ values were not significantly affected by cold stress.

### Group differences in CNS cold response based on fMRI analysis

First-level analysis assessed contrasts associated with cooling relative to warming, and periods of cold relative to periods of neutral skin temperature. Following this, a second-level random effects analysis was performed by grouping subjects based on the amount of activated BAT mass determined by FDG PET imaging during cold exposure (BAT+ vs. BAT− group). In the second level analyses we compared activations between the two groups to identify clusters in core thermoregulatory and interoceptive regions. The identified clusters reflect differential responses between the two groups to body temperature changes during cooling and warming periods. To supplement the spatial profiles of the observed differences in activation, in conjunction we supply the temporal profiles of the smoothed (using non-parametric local regression, LOESS; (Cleveland and Devlin, 1988) activation time series in each of the BAT+ and BAT− groups. These permit an inspection of the differential ranges of the fMRI estimated oscillating neuronal response in the significant regions associated with cooling and rewarming, or cold and neutral. The reporting of results is organized by region.

### Response profiles in the insula

**Figure 4:**
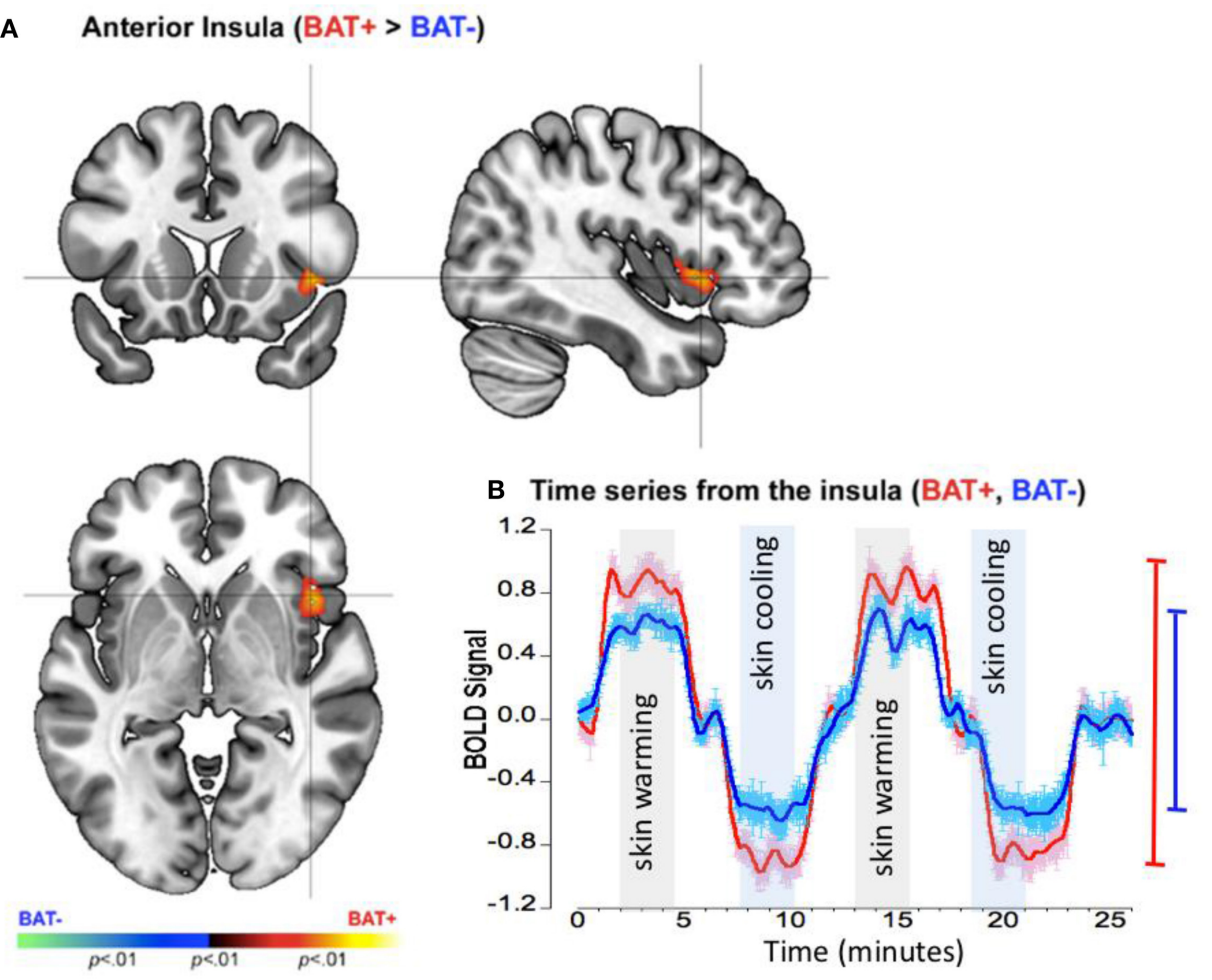
fMRI differences between the BAT+ and the BAT− groups in the insular cortex during periods of skin cooling and warming. Curves are presented as showing the mean value ± standard error of the mean (SEM). **(A)** Clusters of increased activation (Cooling vs. Warming) in the BAT+ group are depicted on an orthoview. **(B)** From this cluster we extracted the first eigenvariate from the modeled fMRI response time series derived from an 18-connected neighborhood of the most significant voxel shown in **(A)**. The smoothed time series (see text) are shown for each of the BAT+ (red curve) and the BAT− (blue curve) groups. Epochs used in the analysis representing skin warming (light gray) and skin cooling (light blue), are clearly denoted. The range bars on the right of the graph depict the range (Max, Min) of the fMRI response in each of the groups (the color scheme is maintained throughout).

As seen in Figure 4A, BAT+ subjects were characterized by a significant increase in the activation profile of the right anterior insula. To examine the basis of this effect, we depict the smoothed time series' across the entire experiment for each of the BAT+ and BAT− groups (Figure 4B).

The time series for the BAT+ group (LOESS: *R*^2^ = 0.643, σ = 0.496, s for residual = 0.497) showed a significantly greater range of the fMRI response between periods of cooling and warming of the skin in the right insula, as compared to the time series for the BAT− group (*R*^2^ = 0.466, σ = 0.491, s for residual = 0.492)(see Table 2). As seen in the range bars, in this region the range of the oscillating response between the cooling and the warming periods was found to be ~40% higher in the BAT+ group when compared to the BAT− group.

**Table 2:** Brain areas showing significant differences in BOLD response between periods of warming and cooling or periods of almost constant neutral and cold temperature observed in the BAT+ group (N_1_ = 12) as compared to the BAT− group (N_2_ = 8).

**Anatomical ROI**		**Critical cluster extent**	**Individual cluster extent**	**Uncorrected *p*-value (*T*-value)**	**Voxel peak (Tal)**
**WARMING > COOLING (Figure 4)**					
Insula	R	136	494	0.002 (3.34)	(44,8,4, BA13)
Midbrain	R	181	705	<0.001 (5.41)	(−2, −24, −4) (Red Nucleus)
Cerebellum	R	181	366	0.001 (3.79)	(14, −22, −36) (R Cerebellar tonsils)
	L	181	237	0.009 (2.62)	(−12, −36, −36) (L Cerebellar tonsils)
**NEUTRAL > COLD (Figure 6)**					
Midbrain	L	112	1,279	<0.001 (4.06)	(−16, −21, −6) (L Subtantia Nigra)
	R	112	1,279	0.001 (3.72)	(9, −24, −10) (R Substantia Nigra)

*R, right; L, left*.

### Response profiles in the mid-brain

**Figure 5:**
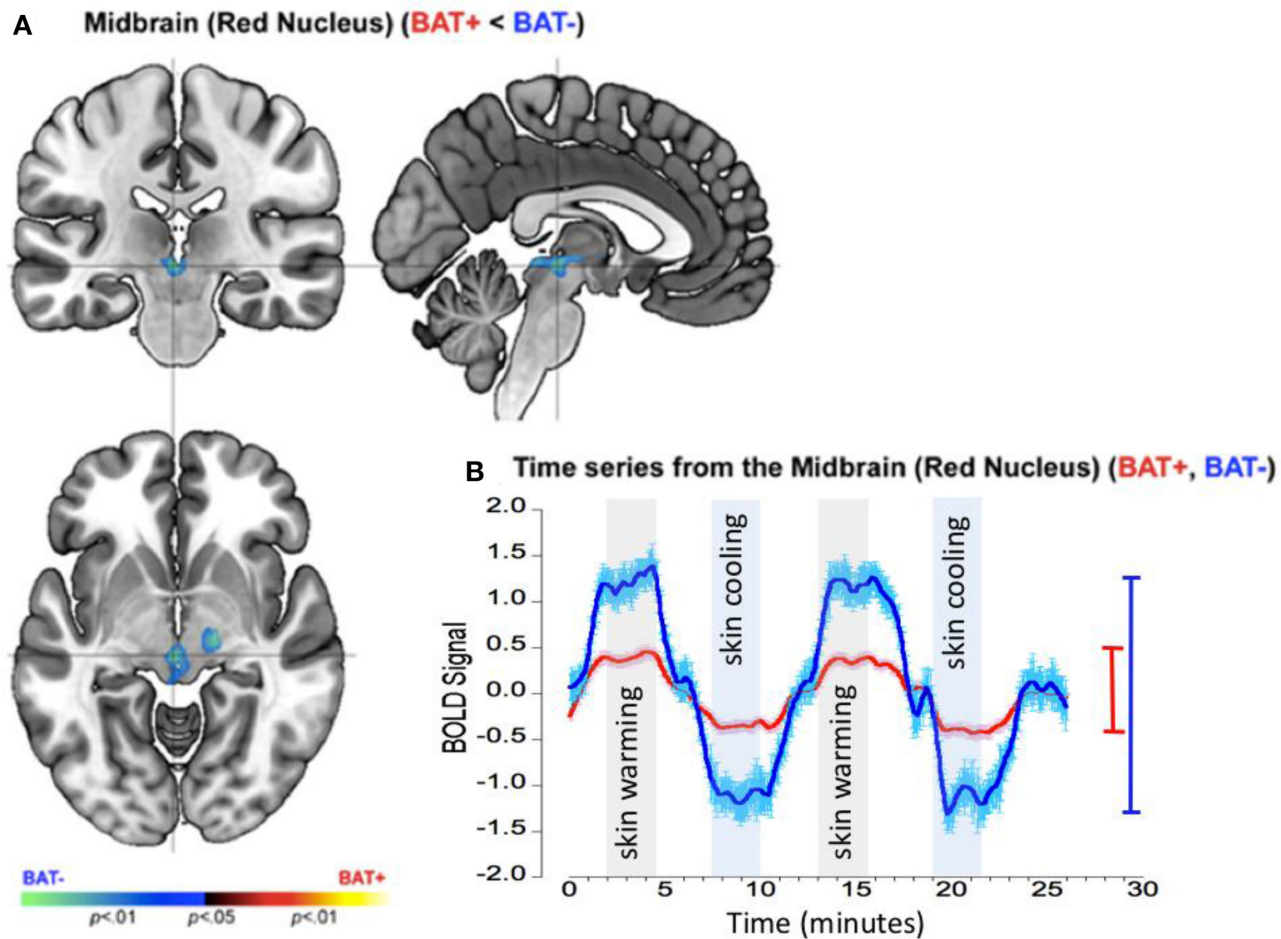
fMRI differences between the BAT+ and the BAT− groups in the mid-brain (specifically the red nucleus) during periods of skin cooling and warming. Curves are presented as showing the mean value ± standard error of the mean (SEM). **(A)** Clusters of decreased activation (Cooling vs. Warming) in the BAT+ group are depicted on an orthoview. **(B)** From this cluster we extracted the first eigenvariate from the modeled fMRI response time series derived from an 18-connected neighborhood of the most significant voxel shown in **(A)**. The smoothed time series (see text) are shown for each of the BAT+ (red curve) and the BAT− (blue curve) groups. Epochs used in the analysis representing skin warming (light gray) and skin cooling (light blue), are clearly denoted. The range bars on the right of the graph depict the range (Max, Min) of the fMRI response in each of the groups (the color scheme is maintained throughout). As seen, the range of the fMRI response is narrower in BAT+, complementing the effects in the insula (Figure 4).

The pattern of responses in the mid-brain complemented those in the insula. In this region the BAT+ group showed significantly *reduced* activations with significant clusters detected at the location of the red nucleus (Figure 5A) and the bilateral cerebellar tonsils. As seen from the accompanying graphs and the range bars, the estimated neuronal response range between periods of skin cooling and warming was ~100% lower in the BAT+ group (*R*^2^ = 0.233, σ = 0.594, s for residual = 0.595) than in the BAT− group (*R*^2^ = 0.292, σ = 1.394, s for residual = 1.395; Figure 5B).

These data suggests two complementary patterns observed in participants with greater sympathetic innervation (BAT+) relative to participants with lower sympathetic innervation (BAT−): (a) BAT+ showed greater CNS sensitivity to skin temperature changes in the right anterior insula but (b) decreased CNS sensitivity of suprapontine midbrain and cerebellar structures. These effects are discussed in greater detail in the Discussion section.

**Figure 6:**
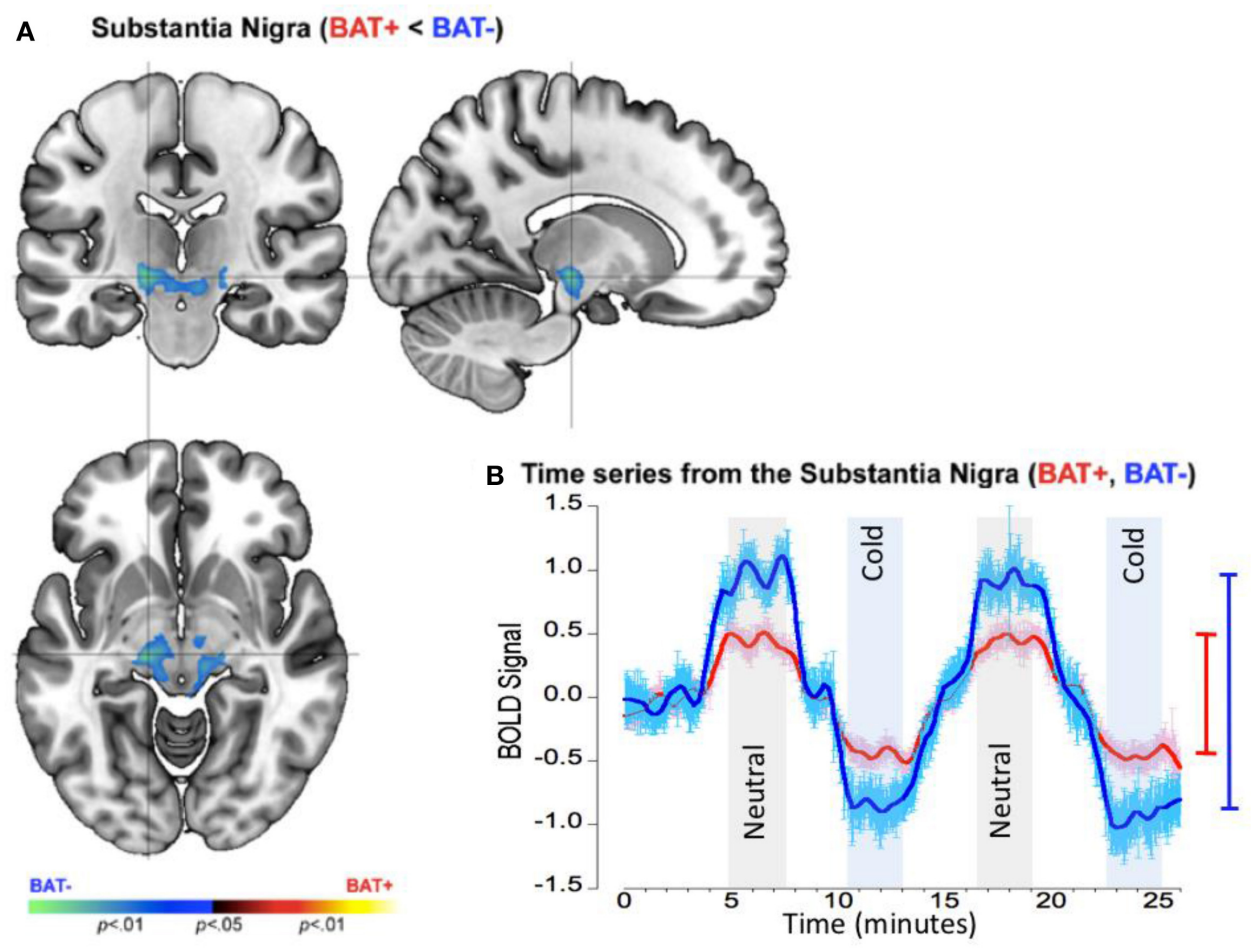
fMRI differences between the BAT+ and the BAT− groups in the mid-brain (approximately the Substantia Nigra) during periods of relative constant skin temperature (cold and neutral). Curves are presented as showing the mean value ± standard error of the mean (SEM). **(A)** Clusters of decreased activation (Cold vs. Neutral) in the BAT+ group are depicted on an orthoview. **(B)** From this cluster we extracted the first eigenvariate from the modeled fMRI response time series derived from an 18- connected neighborhood of the most significant voxel shown in **(A)**. The smoothed time series (see text) are shown for each of the BAT+ (red curve) and the BAT− (blue curve) groups. Epochs used in the analysis representing constant neutral skin temperature (light gray) and constant cold skin temperature (light blue), are clearly denoted. The range bars on the right of the graph depict the range (Max, Min) of the fMRI response in each of the groups (the color scheme is maintained throughout). As seen, the range of the fMRI response is narrower in BAT+, supplements effects observed in the Red Nucleus during cooling and rewarming (Figure 5).

Separate analyses assessed inter-group differences during polar opposite time periods when skin temperature remained stable following adaptation to rewarming (neutral) or cooling (cold). These analyses discovered a single cluster in the substantia nigra (Figure 6A) characterized by a significantly decreased neuronal response range (by ~100%) in the BAT+ group (*R*^2^ = 0.273, σ = 0.574, s for residual = 0.573), relative to the BAT− group (*R*^2^ = 0.455, σ = 0.781, s for residual = 0.782; Figure 6B). As was the case during warming and cooling paradigm, the data suggests that subjects with increased sympathetic innervation show a blunted response to thermal challenge in suprapontine midbrain regions. As we discuss below, these effects may result from inhibitory mechanisms by higher-order brain structures.

## Discussion

Our work demonstrates differences in core interoceptive and regulatory brain areas that effect sympathetic innervation in cold-activated supraclavicular BAT. Differences in neuronal activity between subjects was assessed using co-acquisition of fMRI BOLD signals and skin-temperature changes during an oscillatory thermoregulatory challenge. The applied challenge induced oscillatory and dynamic skin temperature changes during cooling and warming periods, interspersed with periods when skin temperature remained relatively stable (following adaptation as the applied paradigm shifted between cooling and warming).

We restate our CNS results that were obtained comparing subjects with high and low peripheral sympathetic innervation in supraclavicular BAT: (1) The group with increased peripheral sympathetic innervation (BAT+ group) showed increased neuronal activity (and activity range) in the anterior insula during cooling relative to warming of the skin (Figure 4); (2) These effects in the anterior insula were complementary to those observed in the mid-brain (Figure 5); (3) The mid-brain effects were sustained (with slightly different spatial foci) during periods of adapted cold relative to neutral (Figure 6). Individual time-series' for the insula, the red nucleus and the substantia nigra are provided in the on-line “Supplementary Material”.

### Plausible neurophysiological correlates of observed activation foci

It is well established that BAT thermogenesis is mediated by relatively old cytoarchitectonic brain structures that insure autonomic control of BAT; these include most importantly the preoptic anterior hypothalamus (POAH) and various nuclei of the brainstem (Nakamura and Morrison, 2008b; Richard, 2015). Previous rodent studies have demonstrated that midbrain areas, specifically in and around the retrorubral field (an area comprised of A8 dopaminergic neurons caudal and lateral to the red nucleus), exert a tonic inhibitory mechanism on non-shivering thermogenesis (Amini-Sereshki and Zarrindast, 1984; Shibata et al., 1999. These authors showed that procaine (a local anesthetic) microinjections into the retrorubral field region of rats increased both interscapular BAT and rectal temperature in a dose-dependent manner. It was also demonstrated that electrical stimulation of these regions decreased the temperature of interscapular BAT frequency-dependently due to increased enhancement of tonic inhibition. Moreover, these experiments demonstrated that the ability of midbrain neurons to tonically inhibit non-shivering thermogenesis is preserved even after the hypothalamus has been disconnected from the midbrain (through upper midbrain transection). Finally, there is ample evidence that both the substantia nigra as well as retrorubral field neurons project to both the limbic system and cortex via the mesolimbic and mesocortical pathways, which includes the insula (Yetnikoff et al., 2014).

In parallel to these autonomic mechanisms of thermoregulation, humans have developed a repertoire of behavioral responses that regulate body temperature. The motivation for thermoregulatory behavior is the interoceptive experience of thermosensation (the “feeling” of cold) that is under the control of higher brain areas such as the insula, orbitofrontal cortex and superior parietal cortex (Muzik and Diwadkar, 2016). Because the right anterior insula has been shown to contain a sensory representation of small-diameter afferent activity that facilitates discriminative thermal sensation (Craig et al., 2000; Craig, 2002), the observed greater response range of BOLD signal in this interoceptive region of BAT+ subjects suggests higher functional sensitivity of this structure to skin temperature oscillations as compared to that in BAT− subjects. This inference is supported by previous findings that exhibited increased fMRI BOLD signal in this region during menopausal hot flash episodes (Craig, 2002). Additional evidence for the involvement of the right anterior insula in the control of both brainstem and cerebellar regions that are responsible for autonomic cardiovascular response pattern has been documented (Critchley et al., 2000). Thus, the anterior insula emerges as an important node within a broadly hierarchical functional systems that exerts direct or indirect “control” on mid-brain structures that subsequently activate sympathetic responses in the periphery.

Overall, these studies provide the basis for interpreting our results in humans. The observed lower response range of fMRI BOLD signal at the location of the red nucleus/substantia nigra in BAT+ subjects may indicate a higher level of inhibition of these structures by the right anterior insula, which (as discussed above) displayed a higher response range of fMRI BOLD signal to cold in the BAT+ group. Consequently, the observed decrease of midbrain BOLD signal in the BAT+ group can be interpreted as the removal of a tonic inhibitory mechanism on infrapontine brain stem areas (such as the raphe pallidus), resulting in BAT activation through disinhibition-induced stimulation of the central sympathetic nervous system.

### The role of suprapontine midbrain structures during cold

Previous evidence for the involvement of midbrain nigro-striatal structures to thermoregulatory control provides further support for our findings. For example, lesions in rodents at the location of the substantia nigra result in decreased metabolism and hypothermia when exposed to cold, although no deficiencies in thermoregulation were detected at both moderate and hot environmental temperature (Lin et al., 1981). In addition, significant activations of both the red nucleus and the substantia nigra were readily demonstrated in the rat brain during cold exposure (Morimoto and Murakami, 1985), suggesting an active role of these structures in thermal processing. Our results are in agreement with very early reports that demonstrated an inhibitory effect of midbrain structures on the medullary raphe in studies of transected dogs (Keller and McClaskey, 1964). These studies showed that by transecting the connection between the hypothalamus and the midbrain, animals could not keep warm during cold exposure, although they could prevent overheating in a warm environment. The authors interpreted these results as a tonic inhibition of the medulla by the midbrain, which is removed through inhibition of the midbrain by the hypothalamus.

### Cerebellar contribution to cold response

An unexpected result of our study was the detection of cold-induced fMRI BOLD signal differences between the BAT+ and BAT− groups in the cerebellar tonsils. By and large, cerebellar structures have been traditionally viewed as being almost exclusively involved in the planning and initiation of movement. Nevertheless, in recent years cerebellar activations in conjunction with temperature sensation have been detected independently by several investigators (Nunneley et al., 2002; Huang et al., 2011). Interestingly, cerebellar activations at locations very close to the ones reported here were reported based on FDG PET imaging during both cooling and warming paradigms in healthy volunteers (Fechir et al., 2010). Along the same lines, it has been shown that, in addition to coordinating somatic motor activity, the cerebellum regulates visceromotor functions (Onat and Cavdar, 2003; Zhu et al., 2004, 2006), with the dorsomedial, lateral and ventromedial neurons of the hypothalamus (all well-known centers for the regulation of autonomic function). These hypothalamic nuclei project directly to the cerebellum and in turn receive inhibitory signals via cerebellar hypothalamic circuits. Thus, although the exact contribution of cerebellar function to the processing of thermal information is currently uncertain, one can speculate that parts of the cerebellum might be either involved in the acquisition and discrimination of sensory information or might be contributing to motor mechanisms of shivering and thermal muscular tone (Meigal, 2002).

### Difference in DEE between BAT± and BAT− subjects

SNS activity to BAT is regulated by brain regions that process thermal sensation and regulate body temperature in the larger context of whole body energy balance (Richard, 2015). In fact, most circuits controlling BAT thermogenesis play a central role in energy balance regulation (Richard and Picard, 2011; Chechi and Richard, 2015). These circuits include the arcuate nucleus, the POAH as well as the dorsomedial, paraventricular and ventromedial hypothalamic nuclei that exert direct control over a distributed homeostatic regulatory network in the brainstem (Richard, 2015). Given the connection between the regulation of BAT thermogenesis and overall energy balance, the different pattern of DEE response to a relatively short (~2 h) cooling paradigm might be related to the observed differences in thermal interoceptive sensitivity between the two groups.

**Figure 7:**
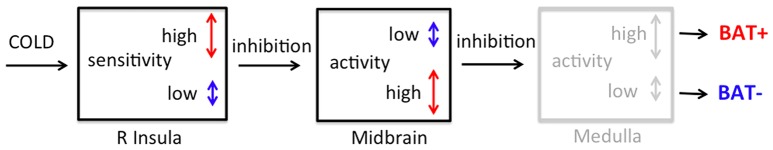
Conceptual model of thermoregulatory control including both interoceptive (R Insula) and autonomic (Midbrain: red nucleus and substantia nigra) brain regions in subjects with high (BAT+) and low (BAT−) sympathetic innervation in supraclavicular BAT. Vertical arrows indicate the magnitude of fMRI BOLD signal change between cooling/warming or cold/neutral conditions for the BAT+ (red) and BAT− (blue) group. As can be seen, BAT+ subjects are characterized by greater magnitude of BOLD signal changes (long red arrow) in the right anterior insula between conditions of skin cooling and warming, as compared to that observed in BAT− subjects (short blue arrow). Subsequently, inhibitory connections between this higher-order interoceptive region and suprapontine brainstem areas induce a narrowing of the neuronal activity range in BAT+ subjects (short red arrow) measured in the rostral midbrain. In contrast, due to the lower dynamic range of neural activity in the right anterior insula of BAT− subjects, the lower inhibitory signal causes a greater magnitude of BOLD signal changes (long blue arrow) in suprapontine brainstem of the BAT− group. These autonomic areas are believed to assert tonic inhibition on infrapontine brainstem regions (e.g., raphe pallidus) when cold response is not needed. Thus, the inhibition of rostral midbrain regions releases tonic inhibition in the medulla (not measured, gray compartment) that leads to increased sympathetic activation of supraclavicular BAT in the BAT+ group.

Taken together, the observed higher sensitivity of a core interoceptive brain area (anterior insula) to cold in the BAT+ group appears to be responsible for the dominant pattern of DEE increases during cold exposure. In contrast, the lower sensitivity of the anterior insula to cold accompanied by continuous tonic inhibition of infrapontine brainstem areas responsible for BAT activation in the BAT− group might be the underlying basis for the observed mixed DEE response during cold. Our data both duplicates as well as provides a rationale for previous findings that showed significant increase in DEE during cold exposure in a BAT+ group but not in a BAT− group (Yoneshiro et al., 2011). Moreover, using a visual analog scale, these authors also showed a higher cold sensation during the summer months in the BAT+ group but not in the BAT− group (Yoneshiro et al., 2016), supporting our observation of increased functional range in core interoceptive brain areas of BAT+ subjects. Collectively, our observations are consistent with a hierarchical system that connects thermal sensation with peripheral response. This system consists of successive levels of inhibitory nodes, where inhibitory signals that originate from the right anterior insula project (possibly via a branch of the mesolimbic pathway) to midbrain areas that normally exert tonic inhibition on the medullary raphe, from where BAT is directly innervated (Figure 7).

### Further considerations and limitations

Because we did not directly measure temperature from *within* the body itself, our CNS effects must be narrowly related to the skin temperature changes measured. Nevertheless, the behavioral relevance of skin temperature changes is profound. It has been argued that skin temperature represents an important auxiliary feedback signal to the main thermoregulatory system, which shortens the system's response time, thereby stabilizing core body temperature (Romanovsky, 2007, 2014). Consistent with this model is the observation that skin temperature is relatively more important for driving most thermoregulatory behaviors (Roberts, 1988), whereas core body temperature is relatively more important for triggering autonomic responses (Jessen, 1981; Sakurada et al., 1993). In the current study, skin temperature was monitored throughout the protocol and the observed skin temperature oscillations were found to be comparable across subjects. Nevertheless, it is possible that differences in both temperature perception as well as in physiological responses to the periodic cooling/warming paradigm exist, even in the recruited highly homogenous group of young lean subjects (age range 20–31 years, BMI 20–25 kg/m^2^). Thus, the possibility for these effects to influence activation cannot be excluded, despite the fact that post-experimental debriefing indicated that subjects perceived the maximum stimulus as “very cold,” but not painful.

Additional limitations of our study are related to some temporal and spatial constraints that are common to nearly all fMRI studies. Our spatial resolution was high relative to many studies (2 × 2 mm in-plane), yet precise anatomical location of sub-cortical nuclei remains difficult due to the small size of the underlying anatomy. Cellular differences in midbrain nuclei are not easily distinguishable using conventional imaging methods, explaining the absence of well resolved anatomical masks. Thus, the designation of the exact anatomical location with respect to the observed significant activations in the midbrain region is challenging and one can only speculate with respect to the exact underlying mechanisms.

A region heavily implicated in thermoregulation by rodent literature, but only infrequently identified in human fMRI studies are the hypothalamic nuclei (Freedman et al., 2006; Diwadkar et al., 2014). This absence (Kochanek and Safar, 2003; Diwadkar et al., 2014) may be attributed to partial volume effects associated with the small structure of the various hypothalamic nuclei and their complementing functionality that includes both excitatory and inhibitory neuronal responses to cold. For example, a likely increased neural activation at the location of the median hypothalamus in the BAT+ group causes decreased activation in the PAOH, which in turn removes inhibition on the dorsomedian hypothalamus, thus increasing its neural activation. It is reasonable to assume that this situation is reversed in the BAT− group. Given the opposing effects of both neuronal activation and deactivation that contribute to the BOLD signal in a relatively small volume, it is not surprising that fMRI BOLD signal changes in the hypothalamic region are difficult to detect.

## Conclusions

Results of our study suggest differential response to cold exposure in interoceptive and regulatory CNS regions between subjects with high and low amount of sympathetic innervation in supraclavicular BAT. The cold-dependent differential response in higher-order interoceptive centers generates varying levels of inhibition over lower-order autonomic midbrain structures, which subsequently regulate sympathetic activation of BAT in an activation-dependent manner. Thus, our data indicates that the high variability of cold-activated BAT mass observed in humans might be, in part, related to different sensitivities of higher-order interoceptive brain regions to skin temperature changes.

## Author contributions

OM and VD jointly designed, performed, analyzed and interpreted the research data.

### Conflict of interest statement

The authors declare that the research was conducted in the absence of any commercial or financial relationships that could be construed as a potential conflict of interest.
