# Supplementary material for: Regulation of Brown Adipose Tissue Activity by Interoceptive CNS Pathways: The interaction between Brain and Periphery
Source: Front Neurosci. 2017 Nov 16;11:640. doi: 10.3389/fnins.2017.00640 (PMC5696740; doi:10.3389/fnins.2017.00640)
Supplement: Supplementary file 1 [file DataSheet1.docx]

Supplemental Data


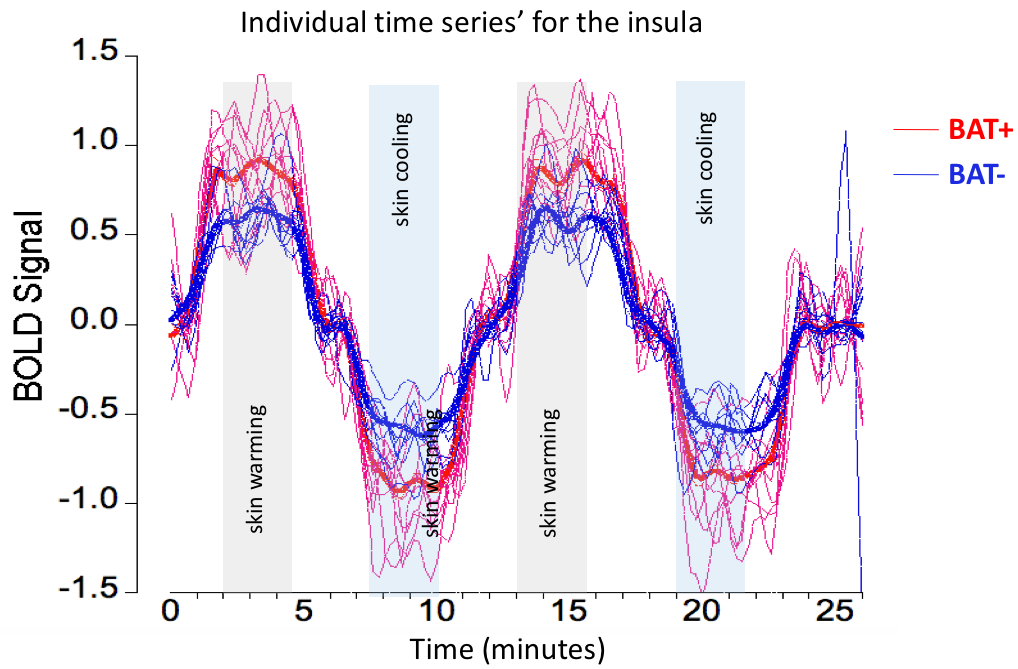


**Figure S1.** Individual fMRI timeseries’ derived from the insula activation locus displayed for subjects in the BAT+ group (thin red lines) as well as for the BAT- group (thin blue lines). In addition, group averaged curves are shown for BAT+ (thick red line) and BAT- (thick blue line) groups.


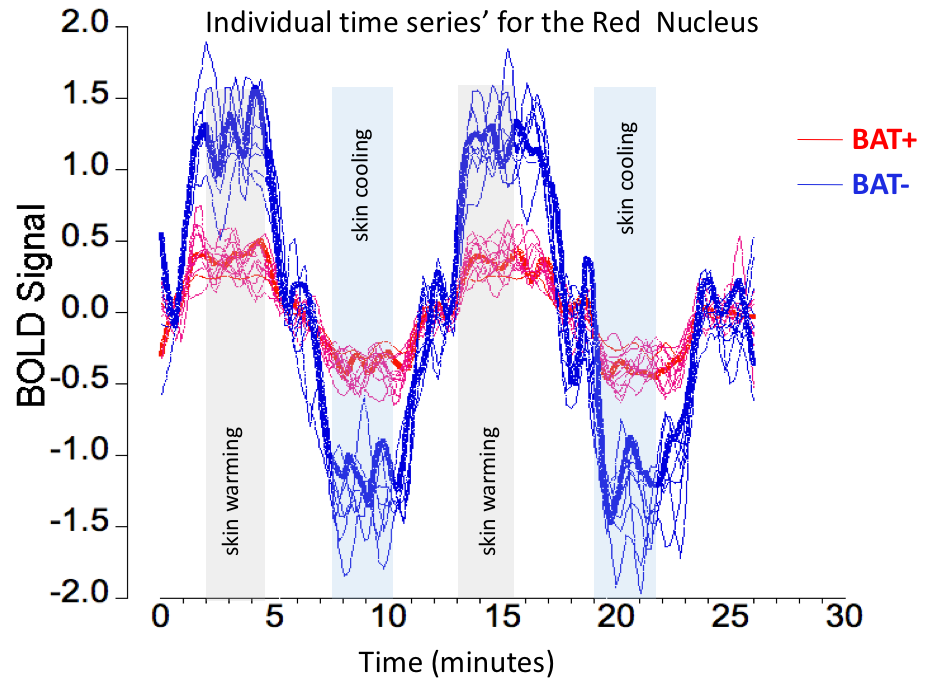


**Figure S2.** Individual fMRI timeseries’ derived from the red nucleus activation locus displayed for subjects in the BAT+ group (thin red lines) as well as for the BAT- group (thin blue lines). In addition, group averaged curves are shown for BAT+ (thick red line) and BAT- (thick blue line) groups.


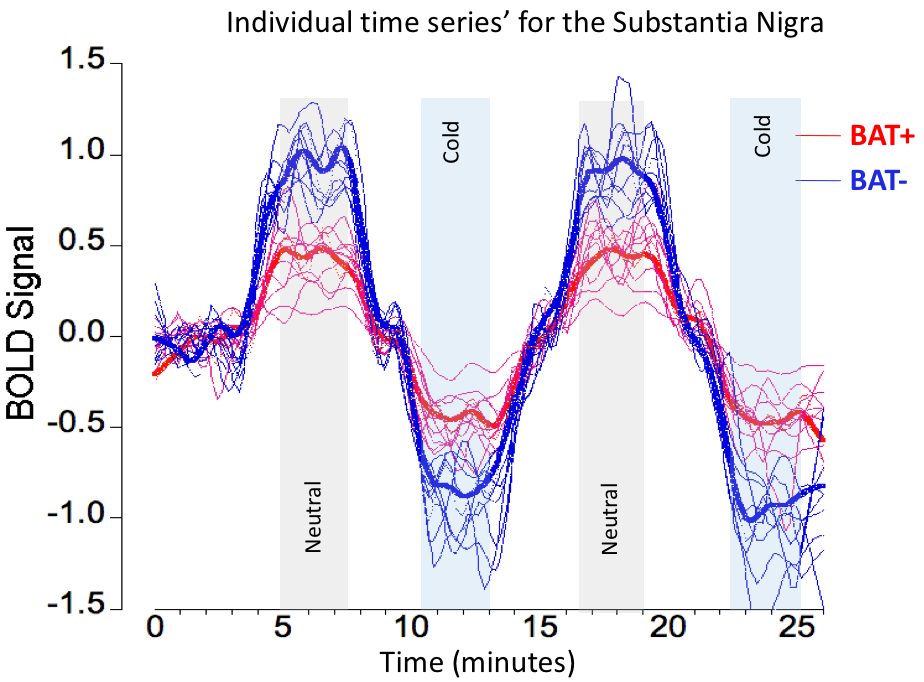


**Figure S3.** Individual fMRI timeseries’ derived from the substantia nigra activation locus displayed for subjects in the BAT+ group (thin red lines) as well as for the BAT- group (thin blue lines). In addition, group averaged curves are shown for BAT+ (thick red line) and BAT- (thick blue line) groups.
